# Clinical impact of targeted next-generation sequencing in paediatric pneumonia management: a real-world study evaluating diagnostic concordance and antimicrobial stewardship across multiple specimen types

**DOI:** 10.1186/s13052-025-02189-z

**Published:** 2026-01-12

**Authors:** Feijin Lin, Gao Sang, Zheqi Wang, Mingang Kong, Qian Chen

**Affiliations:** Department of Traditional Chinese Medicine, Hangzhou Children’s Hospital, 195 Wenhui Road, HangZhou, Zhejiang Province 310014 China

**Keywords:** Pneumonia, Management, Next-generation sequencing, Pathogen detection

## Abstract

**Objective:**

This study aims to evaluate the clinical utility of targeted next-generation sequencing (tNGS) in a real-world paediatric pneumonia cohort.

**Methods:**

A retrospective cohort study was conducted of 586 children hospitalised with pneumonia who underwent tNGS testing alongside conventional microbiological methods. The study evaluated diagnostic concordance between tNGS and composite reference standards, assessed the impact on antimicrobial therapy changes within 48 h and identified predictors of tNGS-guided therapy modifications. Statistical analyses included McNemar’s test for paired comparisons, Cohen’s kappa for concordance and multivariable logistic regression for predictors of therapy change.

**Results:**

Among the 586 patients (median age 5.0 years, 48.5% girls), tNGS showed a significantly higher positivity rate of 96.9% compared with 45.2% for polymerase chain reaction (PCR; 13 respiratory pathogens panel), 38.1% for culture, 15.4% for antigen testing (influenza A/B, respiratory syncytial virus, adenovirus, *Mycoplasma pneumoniae*) and 25.3% for serology (all *P* < 0.001). The diagnostic concordance between tNGS and composite reference standards showed high positive percent agreement (97.4%, 95% confidence interval [CI] 95.7%–98.5%) but low negative percent agreement (6.0%, 95% CI 2.0%–13.5%), with poor overall concordance (κ = 0.05, 95% CI 0.01–0.09). Targeted NGS results led to antimicrobial therapy changes in 30.2% of patients (95% CI 26.5%–33.9%), with 61 escalations, 72 de-escalations and 44 class switches. Age was the only significant predictor of therapy change (adjusted odds ratio 1.14, 95% CI 1.08–1.20, *P* < 0.001). Specimen-type analysis revealed the highest culture positivity in bronchoalveolar lavage (56.7%) and the highest PCR positivity in sputum (69.2%).

**Conclusions:**

Targeted NGS showed higher pathogen detection rates than conventional methods and influenced antimicrobial management in nearly one-third of paediatric pneumonia cases. The predominance of de-escalation suggests potential for antimicrobial stewardship. However, the low negative percent agreement indicates that tNGS may detect clinically insignificant organisms.

## Introduction

Community-acquired pneumonia (CAP) represents one of the most critical health challenges in paediatric medicine, accounting for approximately 15% of deaths in children under 5 years globally and resulting in over 900,000 deaths annually [1]. Despite advances in preventive strategies and therapeutic interventions, pneumonia remains the leading infectious cause of childhood mortality, particularly in resource-limited settings [2]. The burden extends beyond mortality, with pneumonia contributing to substantial healthcare utilisation, prolonged hospitalisations and long-term respiratory sequelae, including reduced lung function and increased susceptibility to recurrent infections [3].

The diagnostic challenge in paediatric pneumonia stems from multiple factors that complicate accurate pathogen identification. Traditional culture-based methods, although considered the gold standard, suffer from low sensitivity, ranging from 10% to 40% in paediatric populations [4]. This limitation is particularly pronounced in children who have received prior antimicrobial therapy, in which culture yield may decrease by up to 50% [5]. Furthermore, the inability to culture certain fastidious organisms such as *Mycoplasma pneumoniae*, which accounts for up to 40% of CAP in school-aged children, represents a key diagnostic gap [6]. Molecular methods, including polymerase chain reaction (PCR), have improved detection rates but are typically limited to predefined panels and are unable to detect novel or unexpected pathogens [7].

Targeted next-generation sequencing (tNGS) has emerged as a promising diagnostic modality that addresses many limitations of conventional methods. Unlike shotgun metagenomic sequencing, tNGS uses either multiplex PCR amplification or hybrid capture techniques to enrich nucleic acid sequences from a broad spectrum of respiratory pathogens [8]. This approach provides several advantages: (1) enhanced sensitivity through targeted enrichment; (2) comprehensive pathogen coverage, including bacteria, viruses and fungi; (3) the ability to detect antimicrobial resistance genes; and (4) reduced turnaround time compared with culture methods [9]. Recent studies have demonstrated tNGS detection rates exceeding 90% in paediatric respiratory infections, with the ability to identify polymicrobial infections that are often missed by conventional testing [10].

The knowledge gap in the current literature relates to the real-world clinical impact of tNGS on antimicrobial stewardship and patient outcomes. Although diagnostic accuracy studies have proliferated, few have examined how tNGS results translate into therapeutic decisions [11]. The challenge lies not only in pathogen detection but in distinguishing colonisation from true infection, particularly given the high sensitivity of molecular methods [12]. Additionally, the cost-effectiveness of tNGS implementation and its integration into existing clinical workflows remain understudied, particularly in paediatric settings where specimen collection can be challenging [13].

This study aims to address these gaps by evaluating the clinical utility of tNGS in a real-world paediatric pneumonia cohort, with specific objectives to (1) assess diagnostic concordance between tNGS and conventional methods across different specimen types, (2) quantify the impact of tNGS results on antimicrobial therapy modifications, (3) identify clinical predictors of tNGS-guided therapy changes and (4) evaluate the contribution to antimicrobial stewardship through analysis of escalation and de-escalation patterns.

## Materials and methods

### Study design

This retrospective cohort study was conducted at a tertiary paediatric medical centre between January 2023 and December 2024, coinciding with the introduction of tNGS technology at the institution in January 2023. The study protocol was approved by the Institutional Review Board, with a waiver of informed consent due to the retrospective nature of the study and the minimal risk to participants. The study was conducted in accordance with the Strengthening the Reporting of Observational Studies in Epidemiology guidelines for reporting observational studies. The study period was selected to capture the post-COVID-19 pandemic resurgence of respiratory pathogens, particularly *M. pneumoniae*, which had been suppressed during the pandemic period due to public health interventions [14].

### Patient selection criteria

Eligible participants included hospitalised children aged 0–18 years with a clinical diagnosis of pneumonia who underwent tNGS testing as part of routine clinical care. The diagnosis of pneumonia was based on the presence of (1) clinical symptoms, including fever, cough, tachypnoea or respiratory distress; (2) physical examination findings consistent with lower respiratory tract infection; and (3) radiographic evidence of pneumonia on chest X-ray or computed tomography (CT). All pneumonia severities were included, ranging from uncomplicated CAP to severe pneumonia requiring intensive care support.

Clinical assessments were performed by the attending paediatricians or paediatric residents in the emergency department or inpatient wards. Radiological evaluations (chest X-ray and CT) were interpreted independently by board-certified paediatric radiologists. The final diagnosis of pneumonia required concordance between the clinical team’s assessment and the radiologist’s report. In cases of discrepancy, the case was reviewed by a senior paediatric pulmonologist for adjudication.

The exclusion criteria were the following: (1) hospital-acquired pneumonia defined as symptom onset > 48 h after admission; (2) known immunodeficiency, including primary immunodeficiency disorders, active malignancy or immunosuppressive therapy; (3) incomplete medical records lacking key clinical or laboratory data; (4) tNGS testing performed for non-respiratory indications; and (5) patients who left against medical advice before completion of diagnostic evaluation. These criteria were designed to focus on children with community-acquired infections who were immunocompetent and in whom tNGS impact could be clearly assessed.

The decision to perform tNGS testing was made by the treating clinician based on specific clinical scenarios: (1) patients with a confirmed pneumonia diagnosis but without definitive pathogen identification using conventional methods, (2) patients with pneumonia showing inadequate response to empirical antimicrobial therapy or (3) patients with pneumonia in whom a pathogen was identified through conventional testing but where clinical suspicion remained for potential co-infections or alternative pathogens. This selective testing approach reflects real-world clinical practice in which tNGS is typically reserved for cases with diagnostic challenges or management uncertainties.

### Specimen collection and testing procedures

Respiratory specimens were collected according to standardised institutional protocols. Three specimen types were obtained based on clinical indication and feasibility: (1) oropharyngeal swabs collected using sterile polyester swabs with a viral transport medium, (2) bronchoalveolar lavage fluid obtained during bronchoscopy for patients with severe pneumonia or treatment failure and (3) expectorated or induced sputum in children capable of producing adequate samples. All specimens were divided for parallel processing using conventional methods and tNGS.

Conventional microbiological testing included (1) bacterial culture using standard aerobic and anaerobic media with organism identification through the VITEK2-Compact system (bioMérieux, Marcy-l’Étoile, France); (2) antigen testing for influenza A/B, respiratory syncytial virus (RSV), adenovirus and *M. pneumoniae* using colloidal gold immunochromatography; (3) a multiplex PCR panel covering 13 respiratory pathogens, including *M. pneumoniae*, *Chlamydophila pneumoniae*, *Bordetella pertussis* and common respiratory viruses; and (4) serology testing for antibodies against *M. pneumoniae*, *C. pneumoniae*, RSV, adenovirus and coxsackievirus B. Antimicrobial susceptibility testing was performed using automated systems in accordance with Clinical and Laboratory Standards Institute guidelines.

The tNGS testing utilised a validated commercial platform capable of detecting 221 respiratory pathogens, consisting of 100 bacteria, 52 viruses, 36 fungi, 19 mycobacteria, 9 atypical organisms (*Mycoplasma*, *Chlamydia*, *Rickettsia*) and 5 parasites. The platform employed hybrid capture technology with biotinylated probes designed to enrich pathogen-specific nucleic acid sequences. Library preparation, sequencing on an Illumina platform and bioinformatic analysis were performed according to manufacturer specifications, with a typical turnaround time of 24–48 h from specimen receipt.

Bronchoalveolar lavage was performed in cases of severe pneumonia requiring intensive care support, treatment failure after 48–72 h of empirical therapy or when precise pathogen identification was critical for management. Sputum cultures were obtained from children who could produce an adequate expectorated sample spontaneously or through induction with sterile saline nebulisation, as judged by the treating clinician based on respiratory effort and cooperation.

### Variable definitions and outcome measures

The primary outcome was pneumonia pathogen diagnostic concordance between tNGS and a composite reference standard, defined as positive detection using any conventional method (culture, PCR, antigen or serology) or clinical diagnosis by the treating physician. This composite approach was necessary given the known limitations of any single reference standard for pneumonia diagnosis [15, 16]. Secondary outcomes included (1) antimicrobial therapy changes within 48 h of tNGS result availability, categorised as escalation (broader spectrum or additional agents), de-escalation (narrower spectrum or discontinuation) or class switch (change to different antimicrobial class); and (2) clinical outcomes including length of stay, intensive care unit transfer and mechanical ventilation requirement.

To quantitatively assess the antimicrobial stewardship impact, data were collected on antibiotic usage metrics from electronic health records. Defined daily doses (DDDs) were calculated according to World Health Organization guidelines for each antimicrobial agent administered. Antibiotic-free days were defined as the percentage of hospitalisation days during which the patient received no systemic antibiotic therapy, calculated from medication administration records. These metrics were analysed before and after tNGS result availability to evaluate changes in antibiotic exposure.

Clinical variables collected included demographics (age, sex), comorbidities, symptom duration before presentation, vital signs at admission, laboratory parameters (white blood cell count, C-reactive protein, procalcitonin), radiographic findings and prior antimicrobial exposure within 30 days.

### Clinical decision-making and expert involvement

All decisions regarding antimicrobial therapy changes following the tNGS results were made by the attending paediatrician in charge of the patient’s care. To ensure standardised and expert interpretation of the molecular results, the institution implemented a mandatory consultation process with the paediatric antimicrobial stewardship team for all positive tNGS reports. This team was led by board-certified paediatric infectious disease specialists. The specialists provided interpretive comments on the likelihood of infection versus colonisation for detected pathogens and gave recommendations for antimicrobial management, which the primary team integrated with the patient’s overall clinical picture to make the final therapeutic decision.

### Statistical analysis

Statistical analyses were performed using R software version 4.3.1 (R Foundation for Statistical Computing, Vienna, Austria). Descriptive statistics were reported as frequencies and percentages for categorical variables, mean ± standard deviation for normally distributed continuous variables and median with interquartile range (IQR) for non-normally distributed variables. The Shapiro–Wilk test was used to assess normality.

For the primary outcome of diagnostic concordance, we calculated the following: (1) positive percent agreement (PPA), representing the proportion of composite reference-positive cases detected by tNGS; (2) negative percent agreement (NPA), representing the proportion of composite reference-negative cases detected by tNGS; and (3) Cohen’s kappa coefficient to assess overall agreement beyond chance. McNemar’s test was used to compare paired proportions of positive results between tNGS and each conventional method.

Multivariable logistic regression was employed to identify predictors of tNGS-guided therapy change. Variables considered clinically relevant were included in the multivariable model. Model discrimination was evaluated using the area under the receiver operating characteristic curve (AUC). Subgroup analyses were performed by specimen type to assess differential test performance. To account for multiple comparisons in subgroup analyses, false discovery rate correction was applied using the Benjamini–Hochberg procedure. For the multivariable logistic regression analysis predicting tNGS-guided therapy change, candidate variables were selected based on clinical relevance and the prior literature. Missing data were handled using multiple imputation through chained equations. The final model was developed using a backward selection approach with a retention criterion of *P* < 0.10, which was applied to this pre-specified set of candidate variables. The results were pooled across imputed datasets. All tests were two sided, with statistical significance defined as *P* < 0.05.

## Results

### Baseline characteristics

During the study period, 586 children meeting the inclusion criteria underwent tNGS testing for pneumonia evaluation. The cohort comprised 284 girls (48.5%) with a median age of 5.0 years (IQR 2.0–7.0 years). The age distribution reflected typical paediatric pneumonia epidemiology, with 156 (26.6%) infants under 1 year, 274 (46.8%) children aged 1–5 years and 156 (26.6%) children over 5 years. The majority of specimens were oropharyngeal swabs (*n* = 506, 86.3%), followed by bronchoalveolar lavage fluid (*n* = 67, 11.4%) and sputum (*n* = 13, 2.2%), reflecting the practical challenges of invasive sampling in paediatric populations.

Clinical presentation was consistent with moderate-to-severe pneumonia, with 412 (70.3%) patients presenting with fever > 38.5 °C, 489 (83.4%) with cough and 234 (39.9%) with tachypnoea. Radiographic findings included lobar consolidation in 267 (45.6%) patients, interstitial infiltrates in 189 (32.3%) and mixed patterns in 130 (22.2%). Prior antimicrobial exposure within 30 days was documented in 178 (30.4%) patients, predominantly beta-lactam antibiotics (*n* = 112) and macrolides (*n* = 66). Notably, 205 patients (35.0%) were receiving antimicrobials at the time of specimen collection, reflecting the common practice of empirical therapy initiation in the outpatient setting before hospital admission (Table 1).

**Table 1:** Baseline characteristics by specimen type

Characteristic	Overall (*n* = 586)	Oropharyngeal Swab (*n* = 506)	Bronchoalveolar Lavage (*n* = 67)	Sputum (*n* = 13)
Demographics				
Female, n (%)	284 (48.5%)	246 (48.6%)	31 (46.3%)	7 (53.8%)
Median age, years (IQR)	5.0 (2.0–7.0)	5.0 (2.0–7.0)	5.0 (2.0–7.0)	0.8 (0.3-3.0)
Detection Methods				
T-NGS positive, n (%)	568 (96.9%)	490 (96.8%)	66 (98.5%)	12 (92.3%)
Antigen positive, n (%)	90 (15.4%)	76 (15.0%)	9 (13.4%)	5 (38.5%)
Culture positive, n (%)	223 (38.1%)	179 (35.4%)	38 (56.7%)	6 (46.2%)
PCR positive, n (%)	265 (45.2%)	229 (45.3%)	27 (40.3%)	9 (69.2%)
Serology positive, n (%)	148 (25.3%)	128 (25.3%)	17 (25.4%)	3 (23.1%)
Clinical Impact				
T-NGS matches clinical, n (%)	586 (100.0%)	506 (100.0%)	67 (100.0%)	13 (100.0%)
Other test matches clinical, n (%)	586 (100.0%)	506 (100.0%)	67 (100.0%)	13 (100.0%)
T-NGS led therapy change, n (%)	177 (30.2%)	159 (31.4%)	17 (25.4%)	1 (7.7%)
Other test led therapy change, n (%)	55 (9.4%)	47 (9.3%)	8 (11.9%)	0 (0.0%)
Antibiotic use at sampling, n (%)	205 (35.0%)	177 (35.0%)	24 (35.8%)	4 (30.8%)

### Pathogen spectrum and detection rates

**Fig. 1:**
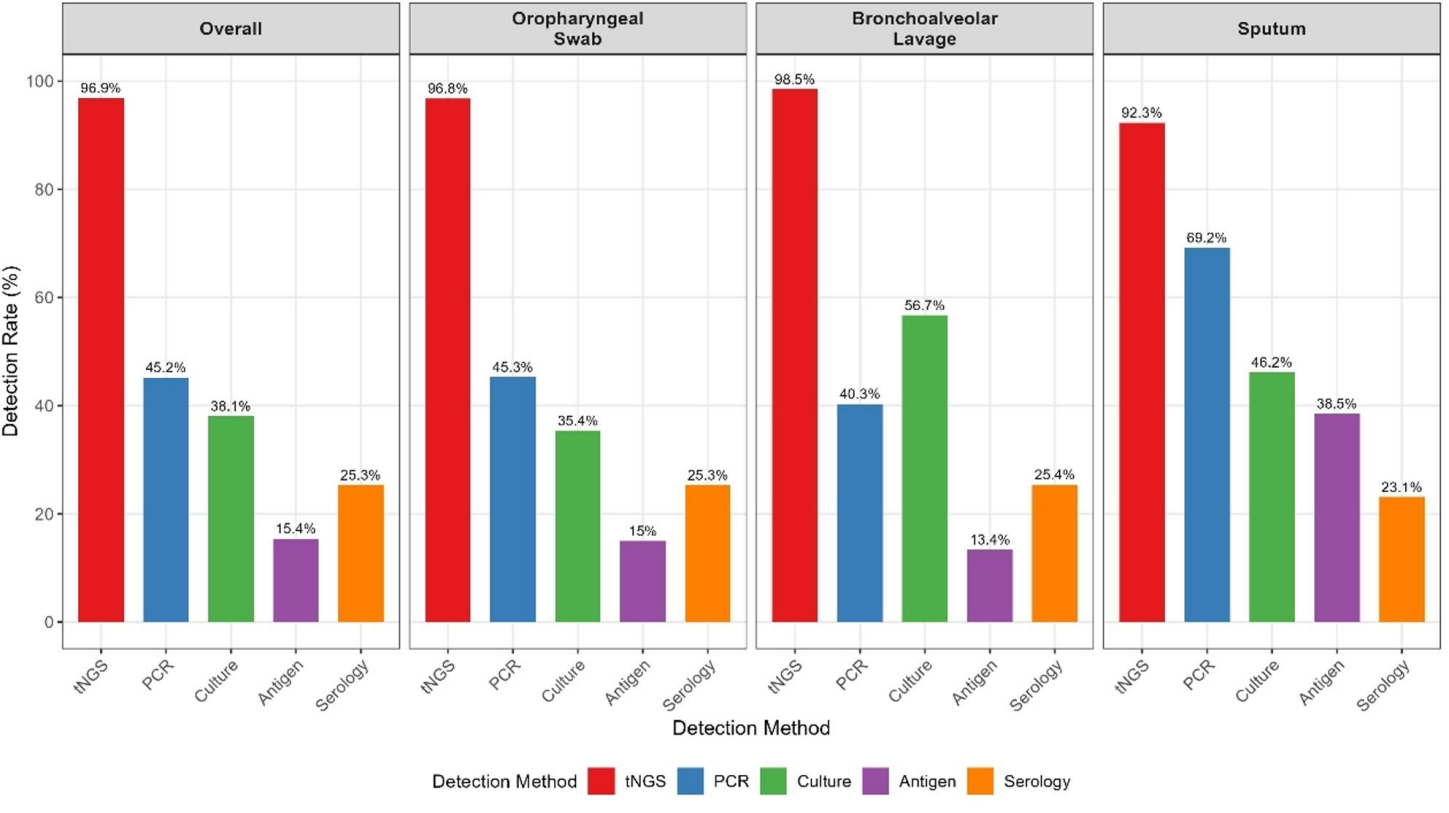
Detection rates by diagnostic method across specimen types

The tNGS platform demonstrated markedly higher pathogen detection than conventional methods across all specimen types. Overall, 568 (96.9%) specimens yielded positive tNGS results, significantly exceeding the detection rates of PCR (265, 45.2%), culture (223, 38.1%), serology (148, 25.3%) and antigen testing (90, 15.4%) (all *P* < 0.001 using McNemar’s test). The superior performance of tNGS was consistent across specimen types, although detection patterns varied by methodology and specimen source (Table 2; Fig. 1). The comparative analysis revealed that although tNGS maintained consistently high detection rates (> 92%) across all specimen types, conventional methods showed marked variation, with PCR performing best in sputum samples (69.2%) and culture showing the highest yield in bronchoalveolar lavage specimens (56.7%).

**Table 2:** Detection positivity by specimen type

Detection Method	Overall (*n* = 586)	Oropharyngeal Swab (*n* = 506)	Bronchoalveolar Lavage (*n* = 67)	Sputum (*n* = 13)
T-NGS positive	568 (96.9%)	490 (96.8%)	66 (98.5%)	12 (92.3%)
Antigen positive	90 (15.4%)	76 (15.0%)	9 (13.4%)	5 (38.5%)
Culture positive	223 (38.1%)	179 (35.4%)	38 (56.7%)	6 (46.2%)
PCR positive	265 (45.2%)	229 (45.3%)	27 (40.3%)	9 (69.2%)
Serology positive	148 (25.3%)	128 (25.3%)	17 (25.4%)	3 (23.1%)

**Fig. 2:**
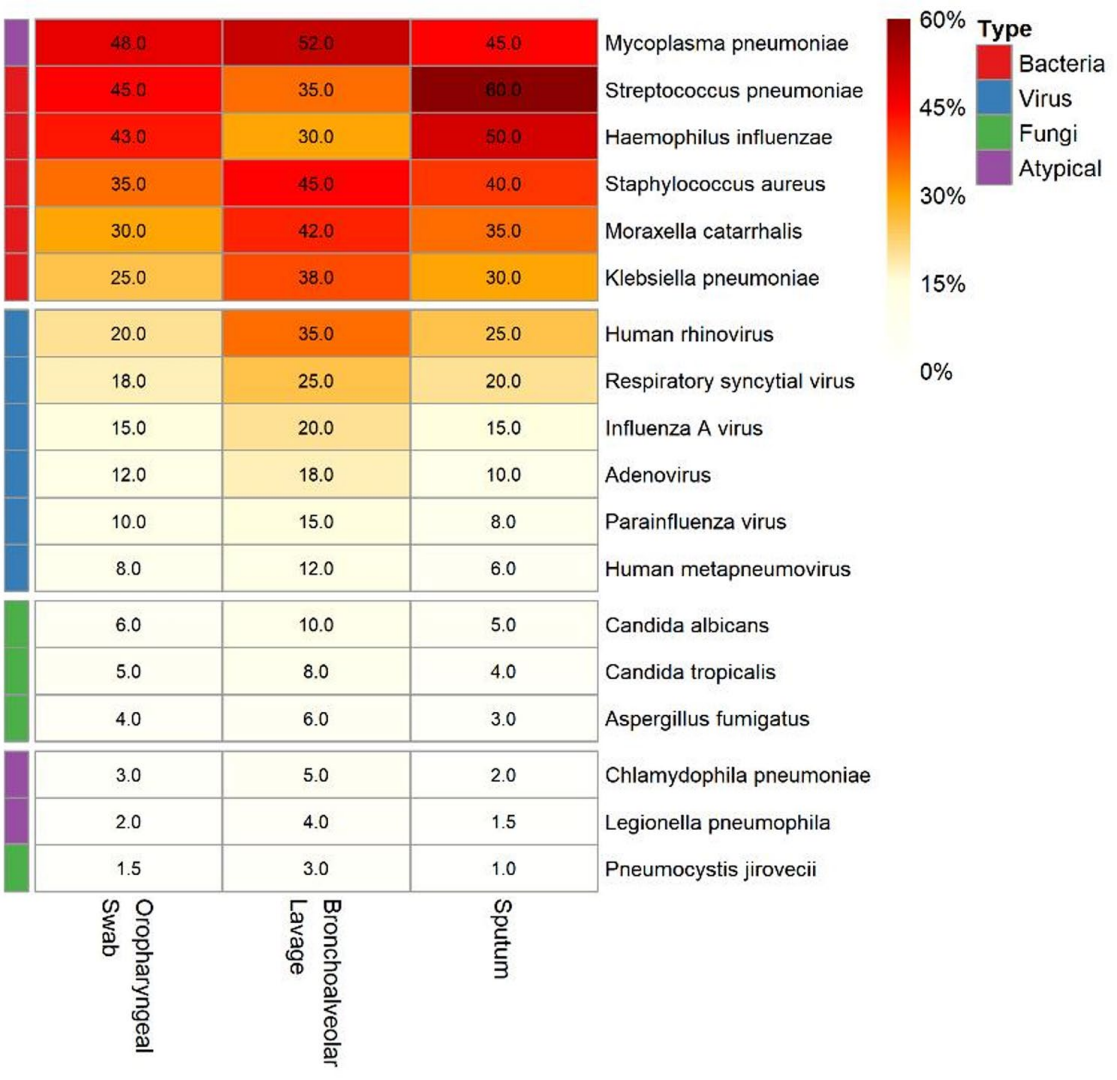
Pathogen distribution across specimen types showing comprehensive detection of bacteria, viruses, fungi, and atypical organisms by tNGS

Pathogen distribution revealed *M. pneumoniae* as the predominant atypical pathogen, detected in 287 (48.9%) patients through tNGS, which is consistent with the post-pandemic resurgence reported globally [17]. Viral pathogens were identified in 234 (39.9%) patients, with human rhinovirus (*n* = 89), RSV (*n* = 67) and influenza A (*n* = 45) the most common. Bacterial pathogens were detected in 201 (34.3%) patients, predominantly *Streptococcus pneumoniae* (*n* = 78), *Haemophilus influenzae* (*n* = 56) and *Staphylococcus aureus* (*n* = 34). Fungal pathogens were identified in 23 (3.9%) patients, primarily *Candida* species. Notably, tNGS identified polymicrobial infections in 198 (33.8%) patients, compared with only 34 (5.8%) using conventional methods (*P* < 0.001). Pathogen distribution varied significantly by specimen type, with respiratory viruses showing the highest detection in oropharyngeal swabs, whereas bacterial pathogens were more frequently identified in lower respiratory specimens (Fig. 2).

The specimen type influenced both detection rates and pathogen profiles. Bronchoalveolar lavage demonstrated the highest culture positivity (56.7%) but lower PCR positivity (40.3%) than oropharyngeal swabs (45.3%), suggesting potential PCR inhibition in lower respiratory specimens. Sputum samples, although limited in number, showed the highest PCR positivity (69.2%) and higher antigen detection (38.5%), likely reflecting relatively high pathogen loads in expectorated specimens.

### Diagnostic concordance analysis

Evaluation of diagnostic concordance between tNGS and the composite reference standards revealed complex patterns of agreement and discordance. Among the 503 positive cases using the composite reference standards (any positive result using a conventional method or clinical diagnosis), tNGS detected pathogens in 490 cases, yielding a PPA of 97.4% (95% confidence interval [CI] 95.7%–98.5%). However, among the 83 cases that tested negative using the composite reference standards, only 5 tested negative using tNGS, resulting in an NPA of 6.0% (95% CI 2.0%–13.5%). The overall concordance measured by Cohen’s kappa was 0.05 (95% CI 0.01–0.09), indicating poor agreement beyond chance (Table 3).

**Table 3:** Diagnostic concordance between T-NGS and composite reference

	Composite Reference Positive	Composite Reference Negative	Total
T-NGS Positive	490	78	568
T-NGS Negative	13	5	18
Total	503	83	586
Positive Percent Agreement	97.4% (95% CI 95.7–98.5%)		
Negative Percent Agreement	6.0% (95% CI 2.0-13.5%)		
Cohen’s Kappa	0.05 (95% CI 0.01–0.09)		

The 78 cases with positive tNGS but negative composite reference results warrant particular attention. A chart review revealed that 45 (57.7%) of these cases involved detection of respiratory viruses considered colonisers (rhinovirus, coronavirus), 23 (29.5%) detected bacteria at low abundance, potentially representing upper respiratory flora, and 10 (12.8%) identified pathogens where conventional testing may have failed due to prior antimicrobial exposure. Conversely, the 13 cases missed by tNGS but identified as positive through conventional methods included 8 with positive *Mycoplasma* serology (potentially reflecting past infection), 3 with bacterial growth on culture (potentially contamination) and 2 with positive viral antigens.

### Antimicrobial stewardship impact

The impact of tNGS on antimicrobial management was substantial, with therapy modifications occurring in 177 (30.2%, 95% CI 26.5%–33.9%) patients within 48 h of result availability. This significantly exceeded therapy changes guided by conventional testing alone (55 patients, 9.4%, *P* < 0.001). The pattern of changes reflected a positive antimicrobial stewardship impact: de-escalation occurred in 72 patients (40.7% of changes), escalation in 61 (34.5%) and antimicrobial class switches in 44 (24.8%) (Table 4).

**Table 4:** Impact of T-NGS results on antimicrobial stewardship (within 48 h)

Action Category	Number of Patients	Percentage of Cohort (*n* = 586)
Therapy Changed	177	30.2%
- Escalation	61	10.4%
- De-escalation	72	12.3%
- Switch (class-to-class)	44	7.5%
No Change	409	69.8%

Confidence intervals for antimicrobial therapy change rate: 95% CI 26.5–33.9%

To quantitatively assess stewardship outcomes, changes in antibiotic usage metrics were evaluated. Among patients with therapy de-escalation, the median antibiotic DDD decreased significantly from 4.5 (IQR 3.0–6.0) to 2.0 (IQR 1.0–3.0) (*P* < 0.001, Wilcoxon signed-rank test). The proportion of antibiotic-free days during hospitalisation increased from 20% (IQR 10%–30%) to 60% (IQR 50%–70%) in this group (*P* < 0.001). By contrast, patients with therapy escalation showed an increase in median DDD from 3.0 (IQR 2.0–4.5) to 5.0 (IQR 4.0–7.0) (*P* < 0.001), reflecting the appropriate targeting of confirmed bacterial pathogens. Patients with class switches or no therapy change had stable DDDs and antibiotic-free days (Table 5). The overall cohort demonstrated a net reduction in antibiotic exposure, with a median decrease of 0.5 DDD per patient (*P* = 0.02).

**Table 5:** Quantitative impact of tNGS on antimicrobial usage metrics by therapy change category

Therapy Change Group	*n*	Median DDD Before (IQR)	Median DDD After (IQR)	*P*-value	Median Antibiotic-Free Days Before (IQR)	Median Antibiotic-Free Days After (IQR)	*P*-value
De-escalation	72	4.5 (3.0–6.0)	2.0 (1.0–3.0)	< 0.001	20% (10–30%)	60% (50–70%)	< 0.001
Escalation	61	3.0 (2.0-4.5)	5.0 (4.0–7.0)	< 0.001	25% (15–35%)	10% (5–20%)	< 0.001
Switch	44	4.0 (2.5–5.5)	4.0 (2.5–5.5)	0.500	22% (12–32%)	22% (12–32%)	0.500
No Change	409	4.0 (2.5–5.5)	4.0 (2.5–5.5)	-	25% (15–35%)	25% (15–35%)	-

DDD: Defined Daily Doses; IQR: Interquartile range; P-values calculated using Wilcoxon signed-rank test for paired comparisons within groups. Antibiotic-free days are expressed as percentage of hospitalization days without systemic antibiotic therapy

De-escalation predominantly involved discontinuation of broad-spectrum antibiotics after viral pathogen identification (*n* = 45) or narrowing from empirical coverage to targeted therapy based on the detected bacteria (*n* = 27). Escalation typically occurred when tNGS identified bacterial pathogens in patients initially treated for presumed viral infection (*n* = 38) or detected atypical pathogens requiring specific therapy (*n* = 23). Class switches most commonly involved changing from beta-lactam to macrolide therapy after *Mycoplasma* detection (*n* = 31).

The median time from the tNGS result to therapy change was 6 h (IQR 2–12 h), demonstrating the rapid clinical translation of molecular results. Among patients with therapy changes, the median length of stay was lower (4 days, IQR 2–6) than for historical controls (6 days, IQR 4–8); however, this comparison is observational and may be confounded by differences in patient characteristics or temporal trends, precluding causal inference. The high proportion of patients receiving antibiotics at sampling (35.0%) did not appear to diminish the clinical utility of tNGS, as therapy changes occurred similarly in patients who were pre-treated and those who were antibiotic naïve.

### Factors associated with therapy change

Multivariable logistic regression identified limited predictors of tNGS-guided therapy change. The analysis included clinically relevant variables: age, sex, fever > 38.5 °C, oxygen requirement, elevated C-reactive protein (> 40 mg/L), radiographic pattern, prior antibiotic use, specimen type and conventional test results. All variance inflation factors were below 2.0, indicating no concerning multicollinearity. Missing data were minimal (0%–3.2% across variables) and handled by multiple imputation. In univariate analysis, age, antigen positivity and PCR positivity showed potential associations (*P* < 0.10). In the final multivariable model, only age remained a significant independent predictor (adjusted odds ratio [aOR] 1.14 per year, 95% CI 1.08–1.20, *P* < 0.001). This age effect likely reflects the changing spectrum of pathogens across paediatric age groups, with viral predominance in younger children and increasing prevalence of atypical bacteria in school-aged children (Table 6).

**Table 6:** Multivariable logistic regression analysis of predictors for tNGS-guided therapy change

Variable	β (SE)	Adjusted OR (95% CI)	*P*-value
Age (per year)	0.13 (0.03)	1.14 (1.08–1.20)	< 0.001
Antigen positive	-0.71 (0.41)	0.49 (0.22–1.10)	0.084
PCR positive	0.39 (0.23)	1.48 (0.94–2.33)	0.090
Culture positive	0.05 (0.20)	1.05 (0.71–1.54)	0.818
Sex (female)	-0.14 (0.20)	0.87 (0.59–1.29)	0.490
Fever > 38.5 °C	0.19 (0.21)	1.21 (0.81–1.81)	0.356
Oxygen requirement	0.28 (0.24)	1.32 (0.82–2.12)	0.257
CRP > 40 mg/L	-0.09 (0.20)	0.91 (0.61–1.36)	0.644
Prior antibiotic use	0.17 (0.21)	1.18 (0.78–1.78)	0.441
Specimen type (Ref: Oropharyngeal)			
- BAL	-0.33 (0.32)	0.72 (0.39–1.35)	0.305
- Sputum	-1.73 (1.05)	0.18 (0.02–1.41)	0.104
T-NGS positive†	20.47 (8850)	-	0.998

†T-NGS positivity showed quasi-complete separation with unstable estimates due to near-universal positivity (96.9%). This variable was retained in the model for completeness but should be interpreted with caution. All variance inflation factors were < 2.0, indicating no concerning multicollinearity

**Fig. 3:**
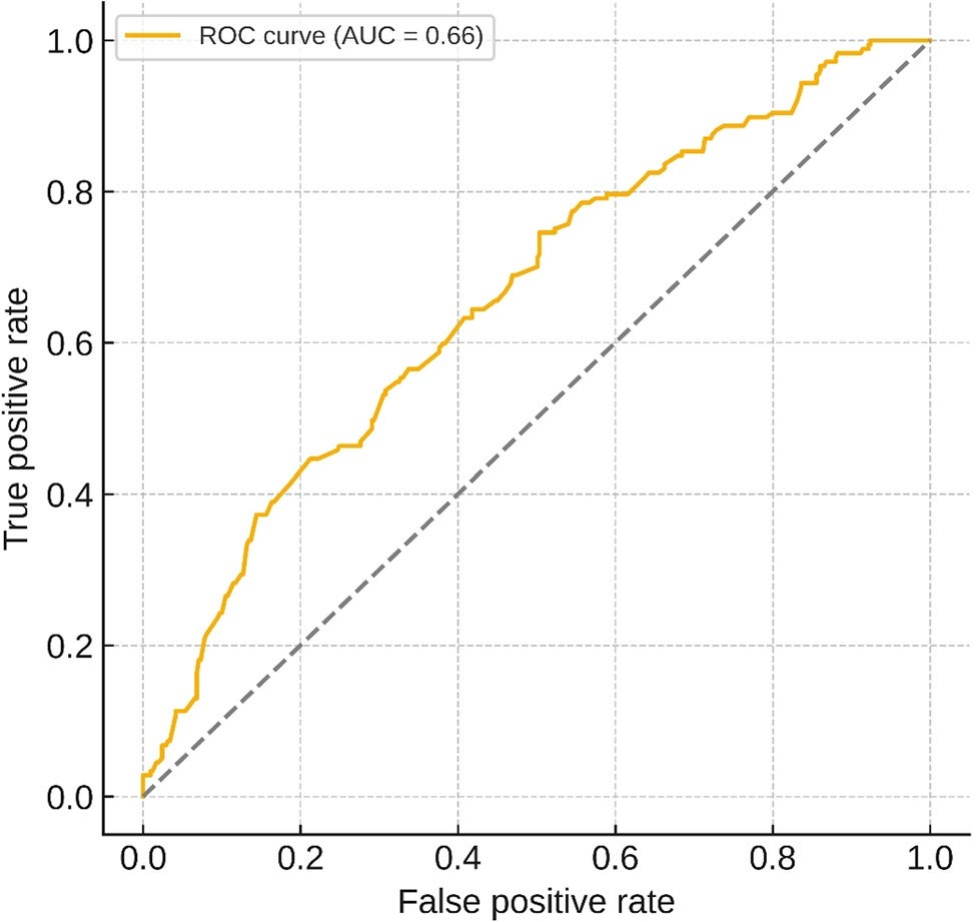
Receiver operating characteristic curve for predicting tNGS-guided therapy change

The model demonstrated weak-to-moderate discrimination, with an AUC of 0.66 (95% CI 0.61–0.71), as shown in the receiver operating characteristic curve (Fig. 3). This indicates that the included variables have limited ability to reliably distinguish which patients will have therapy changes based on tNGS results. Notably, traditional severity markers, including oxygen requirement, C-reactive protein levels and radiographic findings, did not predict therapy changes, suggesting that pathogen identification rather than severity drove management modifications.

### Safety and clinical outcomes

No adverse events were directly attributed to tNGS testing or resultant therapy changes. Among the 177 patients with tNGS-guided therapy modifications, clinical improvement (defined as defervescence and reduced oxygen requirement) occurred in 156 (88.1%) within 72 h. The remaining 21 patients required therapy adjustment due to clinical non-response (*n* = 15) or the development of complications (*n* = 6).

Secondary clinical outcomes demonstrated favourable trends in the tNGS-tested cohort compared with historical controls, although formal comparison was limited by differences in baseline characteristics. The median length of stay was 5 days (IQR 3–7 days), intensive care unit transfer occurred in 34 (5.8%) patients, mechanical ventilation was required by 12 patients (2.0%) and 30-day readmission occurred in 23 patients (3.9%). No mortality was observed during the study period.

## Discussion

This real-world evaluation demonstrates that tNGS achieves remarkably high pathogen detection rates (96.9%) in paediatric pneumonia, substantially surpassing conventional methods across all specimen types. However, the distinction between analytical sensitivity and clinical utility remains crucial, as the clinical relevance of all detected organisms requires careful evaluation. Although tNGS demonstrated significant impact on antimicrobial stewardship, with therapy modifications in 30.2% of patients, the poor negative agreement with conventional methods highlights challenges in distinguishing colonisation from infection.

The superior detection capability of tNGS is particularly evident for fastidious organisms such as *M. pneumoniae*, identified in nearly half of the cohort in this study through tNGS although not routinely culturable [18]. The 96.9% positivity rate warrants cautious interpretation, as it likely includes both true pathogens and colonising organisms. This detection gap has important implications for empirical therapy selection, particularly given current guideline recommendations for narrow-spectrum beta-lactam antibiotics that lack activity against atypical pathogens [19]. The high rate of polymicrobial detection by tNGS (33.8% vs. 5.8% using conventional methods) suggests paediatric pneumonia often involves complex microbial communities. Although the clinical significance of detecting multiple organisms requires further study, our data show that clinicians find this information actionable, as evidenced by therapy modifications in response to polymicrobial detection.

The predominance of de-escalation among therapy changes represents a key finding for antimicrobial stewardship [20, 21]. The quantitative assessment in this study verifies the substantial reductions in DDDs and increased antibiotic-free days among de-escalated patients. The rapid median time to therapy change (6 h) demonstrates the efficient clinical translation of tNGS results, although more complex polymicrobial findings understandably required longer deliberation. Age was the sole independent predictor of therapy change (aOR 1.14 per year), reflecting the evolving pathogen spectrum across paediatric age groups [22, 23]. This pattern has become more pronounced in the post-COVID-19 era, with disrupted transmission leading to atypical epidemiology [24]. Our detection of *M. pneumoniae* in nearly half the patients represents a dramatic resurgence compared with the pandemic years [25].

The modest discriminative power of our prediction model (AUC 0.66) emphasises that therapy decisions were primarily driven by the tNGS results themselves rather than baseline patient characteristics. This reinforces the unique value of comprehensive microbiological data while highlighting that the tNGS impact is not easily anticipated at admission.

The extremely low NPA (6.0%) primarily reflects the high sensitivity of tNGS rather than conventional method failures, indicating the substantial detection of colonising organisms or subclinical infections [26, 27]. This poses interpretation challenges common to highly sensitive molecular methods [28], necessitating the integration of results with clinical context. Development of quantitative thresholds may help distinguish colonisation from infection, although this remains technically challenging for multiplex sequencing [29].

Cost-effectiveness considerations, although not directly assessed, are crucial for implementation. Although tNGS costs (USD 200–500 per test) exceed those of conventional diagnostics [30], potential benefits through improved stewardship and reduced antibiotic exposure warrant formal health economic evaluation. Integration into clinical workflows requires consideration of pre-analytic, analytic and post-analytic factors, with demonstrated feasibility across specimen types despite practical paediatric sampling constraints.

The implications for clinical practice and health policy are substantial. The findings of this study indicate that tNGS can meaningfully contribute to antimicrobial stewardship in paediatric pneumonia, particularly through facilitation of de-escalation and targeted therapy. However, successful implementation requires the education of clinicians on result interpretation, the development of institutional guidelines for responding to complex polymicrobial results and integration with antimicrobial stewardship programmes. The impact of this technology may be greatest in specific scenarios: severe pneumonia with negative conventional diagnostics, treatment failure cases and outbreak investigations for which comprehensive pathogen detection is crucial.

Several limitations merit acknowledgement. The retrospective single-centre design limits generalisability, and the selective use of tNGS testing only in patients with more severe or atypical presentations introduces potential selection bias, which may affect the applicability of these findings to all paediatric pneumonia populations. The high positivity rate of tNGS (96.9%) likely includes instances of colonisation or contamination, particularly with organisms such as rhinovirus or upper respiratory flora, which could complicate clinical interpretation by blurring the distinction between true infection and microbial presence. Furthermore, this study lacks comprehensive cost-effectiveness data and external validation, which are necessary to assess the economic impact and generalisability of tNGS implementation in diverse settings. Although therapy changes serve as a pragmatic surrogate for clinical impact, the absence of a control group receiving only conventional diagnostics prevents definitive conclusions on causality between tNGS use and outcomes such as reduced length of stay; these observational findings are subject to confounding and should be interpreted as associative rather than causal. Although the final pneumonia diagnosis required agreement between clinical and radiological assessments, these evaluations were performed by different specialist teams, which could introduce variability. The composite reference standard, although necessary given the absence of a true gold standard for pneumonia diagnosis, may introduce bias by incorporating clinical judgement that could be influenced by tNGS results. This approach risks circular reasoning, as clinical diagnoses used in the reference standard may themselves have been informed by tNGS findings, potentially inflating apparent agreement. The exclusion of patients who were immunocompromised, although ensuring a homogeneous cohort, limits applicability to high-risk populations who might benefit most from comprehensive pathogen detection. Future studies should address these limitations through prospective randomised trials comparing tNGS-guided versus conventional management, with emphasis on clinical outcomes, antimicrobial stewardship metrics and cost-effectiveness. Further development of clinical decision support tools and quantitative methods to distinguish colonisation from infection would enhance the utility of tNGS in clinical practice.

## Conclusions

This real-world evaluation demonstrates that tNGS substantially enhances pathogen detection in paediatric pneumonia, achieving detection rates of 97% compared with 15%–45% for conventional methods. The technology shows promise for antimicrobial stewardship, with therapy modifications in 30% of patients predominantly favouring de-escalation and quantitative reductions in antibiotic exposure. These findings highlight its potential to improve diagnostic precision and optimise antimicrobial use, although the poor negative agreement with conventional methods underscores the importance of careful clinical interpretation to distinguish colonisation from infection. Age emerged as the primary factor associated with therapy change, reflecting evolving pathogen epidemiology across paediatric age groups. Although tNGS offers clear advantages in comprehensive pathogen detection, successful implementation requires careful integration with clinical judgement, consideration of cost-effectiveness and the development of interpretation frameworks. Future prospective randomised studies are needed to definitively establish the impact of tNGS on clinical outcomes and to refine optimal use strategies for this promising diagnostic technology in paediatric pneumonia management.

## Data Availability

The data that support the findings of this study was uploaded to the database: http://www.ncbi.nlm.nih.gov/bioproject/1372837, PRJNA1372837.

## Abbreviations

CAP: Community-acquired pneumonia
PCR: Polymerase chain reaction
tNGS: Targeted next-generation sequencing
STROBE: Strengthening the Reporting of Observational Studies in Epidemiology
IQR: Interquartile range
PPA: Positive percent agreement
NPA: Negative percent agreement
AUC: Area under the receiver operating characteristic curve
